# Development of a surface tension mediated technique for dry stabilization of mammalian cells

**DOI:** 10.1371/journal.pone.0193160

**Published:** 2018-03-05

**Authors:** Jason Solocinski, Quinn A. Osgood, Eric Rosiek, Lukas Underwood, Oleg Zikanov, Nilay Chakraborty

**Affiliations:** 1 Department of Mechanical Engineering, University of Michigan-Dearborn, Dearborn, Michigan, United States of America; 2 OtziBio LLC, Livonia, Michigan, United States of America; University of Maryland, UNITED STATES

## Abstract

Dry state preservation at ambient temperatures (lyopreservation) is a biomimetic alternative to low temperature stabilization (cryopreservation) of biological materials. Lyopreservation is hypothesized to rely upon the creation of a glassy environment, which is commonly observed in desiccation-tolerant organisms. Non-uniformities in dried samples have been indicated as one of the reasons for instability in storage outcome. The current study presents a simple, fast, and uniform surface tension based technique that can be implemented for lyopreservation of mammalian cells. The technique involves withdrawing cells attached to rigid substrates to be submerged in a solution of lyoprotectant and then withdrawing the samples at a specific rate to an inert environment. This creates a uniform thin film of desiccated lyoprotectant due to sudden change of surface tension. The residual moisture contents at different locations in the desiccated film was quantified using a spatially resolved Raman microspectroscopy technique. Post-desiccation cellular viability and growth are quantified using fluorescent microscopy and dye exclusion assays. Cellular injury following desiccation is evaluated by bioenergetic quantification of metabolic functions using extracellular flux analysis and by a Raman microspectroscopic analysis of change in membrane structure. The technique developed here addresses an important bottleneck of lyoprocessing which requires the fast and uniform desiccation of cellular samples.

## Introduction

Storage of biologics and cellular material using lyopreservation has the potential to simplify logistics and transportation by reducing the need for cold-chain logistics. Development of such a technique for mammalian cells can have a significant impact in clinical application of advanced cell-based therapies, particularly in resource limited regions [1, 2]. The success of lyopreservation has been theorized to rely upon the creation of a high viscosity extra and intracellular environment at an advanced state of desiccation, where low molecular mobility prevents any degradative reactions [3, 4]. This mechanism of preservation is frequently observed in nature among a wide variety of bacteria [5], animals, and plants (anhydrobiotes) [6], suggesting that this ability, developed by ancient cell types, may have been a critical factor of successful colonization of terrestrial earth [7, 8].

Lyopreservation is believed to involve use of glass forming agents, such as trehalose, during acute desiccation to impart stability to the biomolecules. There are two important obstacles related to successful lyopreservation of mammalian cells: first being overcoming the processing injury for the cells, followed by storage in desiccated state. The principal concerns which must be considered for lyopreservation are imparting desiccation tolerance [9], creating a uniformly desiccated result [10], and inhibiting other associated cell injury mechanics such as cumulative osmotic stress [11].

While it is important to explore techniques to increase desiccation tolerance of cells using different chemical strategies, it is important to develop strategies to desiccate mammalian cells that minimize cellular injury [12]. Injury during lyoprocessing may result from the inherent sensitivity of mammalian cells to osmotic stress and non-uniformity of the samples during dry processing [10]. Fast desiccation techniques which limit exposure of cells to high osmotic stress and improve uniformity in residual moisture content have been proposed to be critical for developing successful lyoprocessing methods [10, 13].

In this study, we have developed a surface-tension mediated fast drying technique that can be used to desiccate mammalian cells attached to a substrate with highly uniform residual moisture content. When cells attached to glass substrate are withdrawn from a solution of lyoprotectant to an inert environment, the sudden change of surface tension creates a uniform thin film of uniformly desiccated lyoprotectant. Commonly known as dip-coating, the technique is widely applied for ultraclean drying of semiconductor substrates in the electronics industry [14–16].

A Raman microspectroscopic technique was used to determine both residual moisture content as well as spatial uniformity of the lyoprocessed samples. Analysis of the desiccated samples indicate significantly low moisture content as well as high spatial uniformity. Viability and bioenergetic assays were to demonstrate post-desiccation cellular health. Finally, a detailed microspectroscopic study of the change in chemical composition of the cell membrane was undertaken to investigate the injury to the cellular membrane structure or composition.

## Materials and methods

### Cell culture and trehalose internalization

Human hepatocellular carcinoma (HepG2) cells were cultured in Opti-MEM (Gibco, Carlsbad, CA) supplemented with 5% fetal bovine serum (Gibco) and penicillin-streptomycin (100 units/mL penicillin G, 100μg/mL streptomycin sulfate; HyClone-Thermo Scientific, Logan, UT) under standard cell culture conditions (95% air, 5% CO_2_, and 37°C). Cells were maintained at a confluency of 90% using 75 cm^2^ cell culture flasks (Corning Incorporated, Corning, NY). Cells were collected using trypsinization followed by centrifugation. A hemocytometer (Hausser Scientific, Horsham, PA) was used to count live cells using Trypan Blue exclusion technique. Collagen coated microscope slides (Fisher Scientific, Pittsburg, PA) were outfitted with FastWell^™^ reagent barriers (Grace Biolabs, Bend, OR) to create a defined cellular boundary on the glass substrate and prevent any non-standard attachment conditions. The cell suspension was then pipetted into the wells at a final concentration of 150k cells/well.

It has been previously shown that internal trehalose benefits mammalian cells during desiccation processes. Therefore trehalose was internalized in the intracellular space via phase endocytosis [9]. Cells were incubated with growth media supplemented with 200 mM trehalose (Ferro Pfanstiehl Inc., Waukegan, IL). Under similar condition, HepG2 cells have been shown have an intracellular concentration in excess of 2.5 mM following fluid phase endocytosis [17].

### Surface-tension based lyoprocessing technique

**Fig 1:**
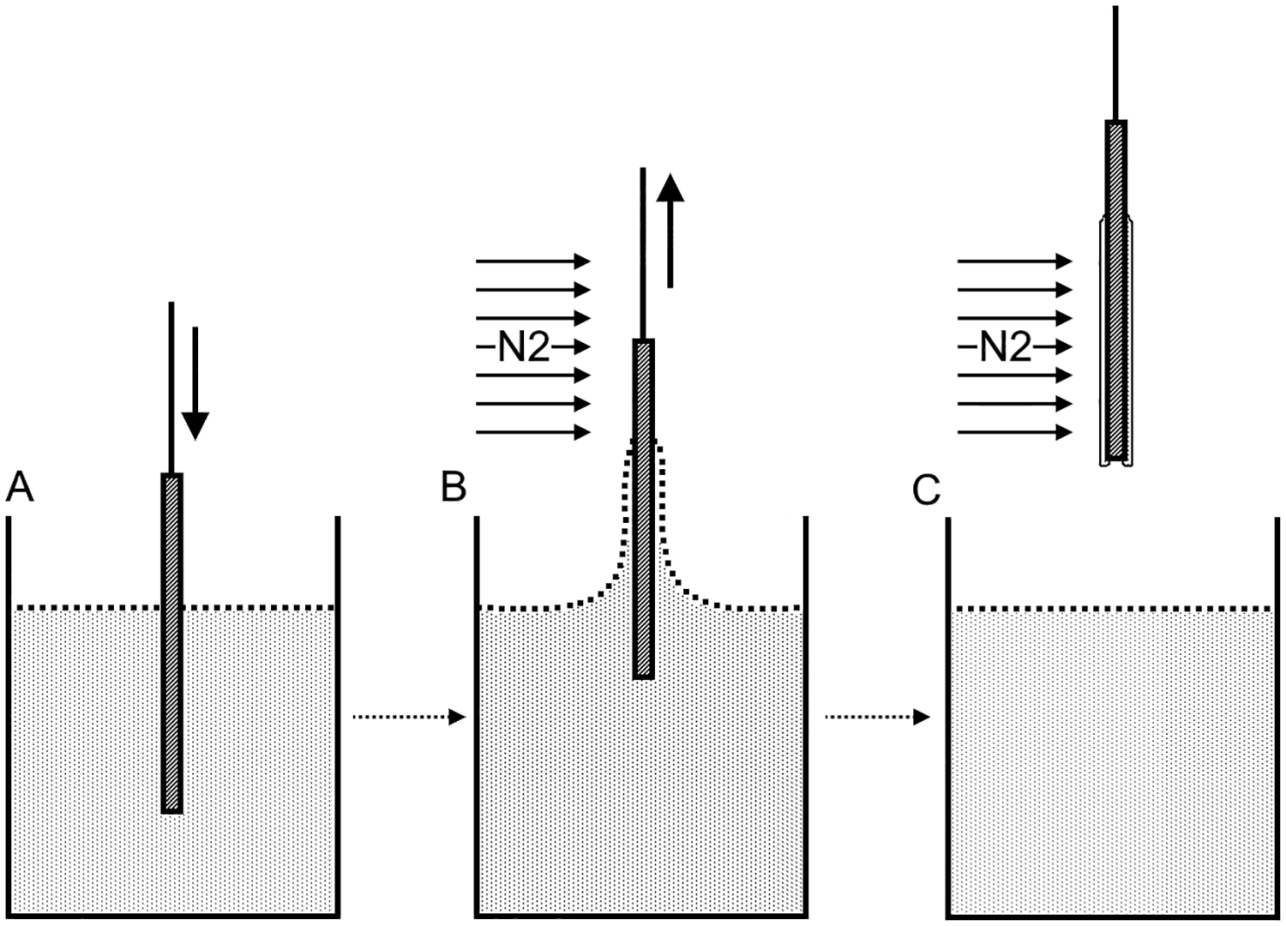
Diagram illustrating the surface tension based lyoprocessing technique. A) The substrate is immersed into a trehalose based lyoprotectant solution (indicated as the dotted region). B) The substrate is withdrawn in an inert environment over a stream of Nitrogen gas at a prescribed drawing rate, creating a desiccated film. C) The desiccated film is shown as a very thin layer attached to the substrate.

The technique involves use of sudden change of surface tension to create a thin film of desiccated lyoprotectant solution when cells attached to a glass substrate is withdrawn into an inert environment from submerged condition (Fig 1). A stepper motor based drawing assembly is used to withdraw the substrate with attached cells at specific rates (50–200 mm/min). A trehalose-based solution [13] is used as lyoproetctant and an active flow of high purity nitrogen gas at 10 psi is used to create the inert environment. The process is stopped once the entire slide is in the inert environment.

### Membrane integrity and long-term viability

Following lyoprocessing the cells using the mechanism described above, the membrane integrity analysis was performed using Syto-13/ ethydium bromide membrane integrity assays (Thermofisher Scientific). The stock solution for Syto-13/ethydium bromide staining was prepared by adding 10 mL of 1 mg/mL Syto-13 solution (aq.) and 5 mL of 1.0 mg/mL ethydium bromide solution (aq.) to 8 mL of OptiMEM (Thermofisher Scientific). After rehydration, 100 μL of Syto-13/ethydium bromide solution was added to the cells attached on coverslips, and the samples were incubated at 37°C for 5 min. These samples were then imaged using an inverted microscope (Olympus BX51, Olympus Inc. Japan) with FITC and PI filters. Cell membrane integrity was determined immediately after rehydration by counting intact membranes (green) and membrane compromised (red) cells in five representative images obtained at different locations on the slide.

Long-term viability and growth profiles were determined by incubating the rehydrated cell samples in fully complemented medium for 7 days in 100 mm culture dishes. Parallel samples were used for the various experimental treatments, and the viability of cells after desiccation was measured by counting cells on days 1, 3, and 7. To ensure that only viable cells were counted, the membrane integrity of these cells was determined by trypan blue exclusion.

### Metabolic profile

Analysis of metabolic function for both dip-coated and unprocessed cells were carried out on an extracellular flux analyzer (XFp Seahorse, Agilent Technologies, Santa Clara, CA). Following dip-coating, the cell samples were rehydrated immediately in growth medium and cultured for 14 days. Then cells were trypsinized and counted using trypan blue exclusion. Cells were seeded in prescribed microwell plates at a seeding density of 2x10^4^ cells per well. Both oxygen consumption rate (OCR) (representing mitochondrial respiration) and extracellular acidification rate (ECAR) (representing glycolysis) measurements were taken using standard Seahorse protocol, following optimization. Briefly, XFp cartridge sensors were hydrated and injection ports loaded by the following; mitochondrial test reagents used oligomycin (1 μM), carbonyl-cyanide-4-(trifluoromethoxy) phenylhydrazone (FCCP, 0.5 μM), and rotenone/antimycin A (0.5 μM). Glycolysis test reagents used D-glucose (10 mM), oligomycin (1 μM), and 2-deoxyglucose (2-DG, 50 mM). All reagents have corresponding final concentrations in cell chambers immediately after being listed. Oligomycin was used as the F_0_F_1_-ATPase inhibitor and oxygen consumption rates measured in presence of this inhibitor indicate proton leak. FCCP acts as an uncoupling agent that collapses the mitochondrial proton gradient and thereby uncouples oxidation from phosphorylation, maximizing oxygen consumption rates. The contribution of energy generation via non-mitochondrial processes was quantified in relation to the overall oxygen flux using rotenone and antimycin A. Rotenone and antimycin A inhibit complex I and III, respectively, of the respiratory system. Glucose was supplied as to fully saturate basal ECAR. Then, since oligomycin inhibits ATP synthase in the electron transport chain, ATP/ADP ratio decreases, which stimulates glycolysis. 2-DG is a glucose analog which competitively inhibits hexokinase, effectively arresting glycolysis.

### Raman microspectroscopy

Raman microspectroscopic measurements for both residual water content and membrane function were conducted with a customized confocal microscope and Raman spectrometer combination (UHTS 300, WITec Instruments Corp., Germany). Raman spectra were collected using an EMCCD camera (Andor Technology, UK) at a spatial resolution of 500 nm. A 532 nm solid state laser calibrated to 20 mW was used for excitation and spectra were collected using a custom 50X high resolution objective (Mitutoyo, Japan).

#### Evaluation of spatial uniformity and residual moisture in desiccated samples

A Raman microspectroscopy technique was used to correlate residual moisture content in desiccated samples. The desiccated samples were transferred to a custom made sealed quartz chamber immediately following dip-coating at the four drawing rates using 1.2 M trehalose solution. This was done with a view to minimize the effect of interference from atmospheric humidity. The trehalose films were then scanned at arbitrarily preselected points to acquire average spectrum for each condition. A sample processed at 100 mm/min was analyzed by a linear Raman scanning method across a region of 22 mm, with a linear spectra polling rate every 30 μm. Raman signatures were collected from a location close (2–5 μm) to the glass substrate. The ratio of OH stretching peak (~3430 rel. 1/cm) to a characteristic trehalose peak (C-O-H bending peak, 923 rel. 1/cm) was used to quantify residual water. Such spectroscopic data collected from different spatial locations was used to profile uniformity of the residual water content in desiccated samples.

Solutions of trehalose buffer in water were created on a weight by weight basis ranging from 0 to 55%. A 100% sample was created by drying a 55% droplet in a 210°C oven for 24 hr. Samples were scanned via Raman spectroscopy to generate a calibration curve relating the spectral intensity of water to trehalose (I_3360_:I_923_) and the water content (g_H20_/g_DW_), gravimetrically obtained.

Samples of each of the four conditions were also scanned to determine the residual moisture content immediately after processing, after 2 hr in a desiccation box containing CaSO_4_ (Drierite, WA Hammond, Xenia, OH) and after 18 hr in a vacuum (-25 in. Hg) oven at 210°C (S1A Fig). This was done to verify the extent of desiccation reached by the lyoprocessing technique described here.

#### Molecular analysis to monitor changes in membrane composition

The changes in the membrane composition were analyzed using a hyperspectral molecular analysis technique. Raman spectral arrays were acquired for unprocessed cells and for cells that was desiccated using the process described here. Membrane composition for drawing speeds of 50 mm/min and 100 mm/min were compared and evaluated after maintaining the rehydrated cell samples in culture condition following desiccation processing. Hyperspectral maps were collected using a spatial dimension of 40μm×40μm with a spectral dimension of 80x80 pixels. Each array of scans was collected using an integration time of 0.5 s. Principal component analysis (PCA) was used to reduce the dimensionality of the collected spectral data arrays into principal components (PCs). A non-linear iterative partial least squares algorithm was used for related vector analysis to eliminate noise in the spectral baseline [18]. Following noise reduction using PCA, hierarchal cluster analysis was used to construct an average spectral signature for the cell membrane of the HepG2 cells. Substrate and water spectral contributions were subtracted at this point. A detailed deconvolution analysis was performed to find structural relationship with the functional groups and proteins in the cell membrane using Peakfit (V4, Systat San Jose, CA). The deconvolution analysis was done for the CH2 asymmetric stretching and amide III peaks, located at approximately 2940 and 1320 rel. 1/cm, respectively. The amide III peak at 1320 rel. 1/cm is contained in the protein fingerprint region of Raman spectra and correlates to α-helical secondary protein structures and is known to decrease with protein denaturation [19]. Both CH2 asymmetric and CH2 symmetric have similar forms but the asymmetric band was chosen due to less variation in the dataset. Structural composition of the cell membrane was evaluated by taking the ratio of the CH2 asymmetric peak intensity to that of the amide III peak.

### Statistical analysis

Data were analyzed with a one-way analysis of variance (ANOVA) on ranks of experimental groups. Origin Pro 2017 (Northampton, MA) was used for the analyses. Data sets are presented as mean ± (SD).

## Results

### Estimation of spatial uniformity and residual moisture analysis

**Fig 2:**
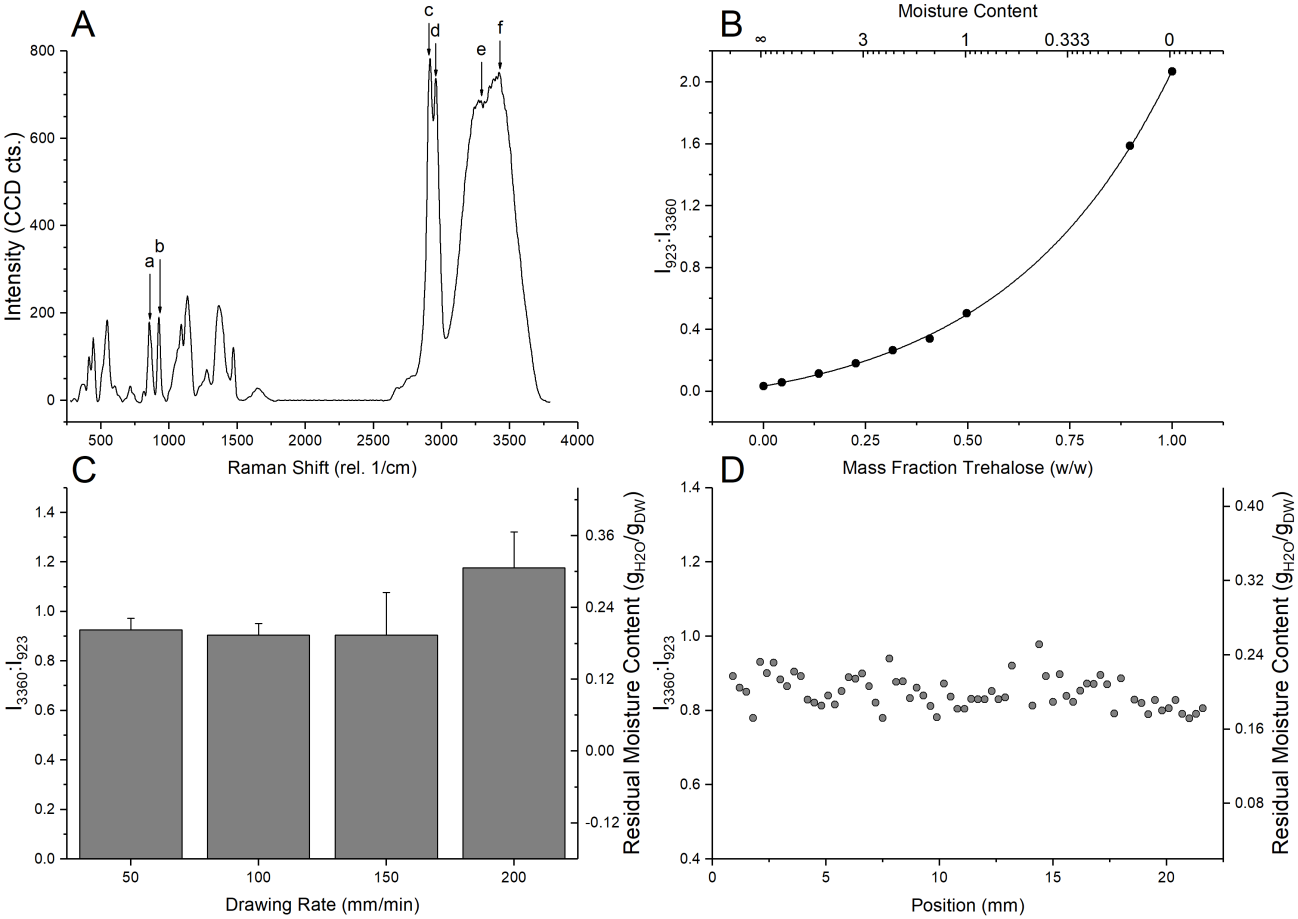
Residual moisture analysis using Raman microspectroscopy. **A)** Representative Raman spectrum of a 1.2 M trehalose-water binary solution. Significant peaks are detailed in Table 1. **B)** Correlation of intensity ratios and residual water content by weight of standardized trehalose solutions. **C)** Average water proportions for samples processed at 50–200 mm/min. **D)** Distribution of Raman spectral ratios and water content for water (3430 rel. 1/cm) and trehalose (923 rel. 1/cm) across a large area for an acellular sample processed at 100 mm/min with a 1.2 M trehalose solution.

The spatial uniformity of the dry-processed samples was quantified using a Raman microspectroscopic technique as described before. A trehalose concentration of 1.2 M was used in the desiccation solution as lesser molarities produced poorer cellular viability outcomes upon rehydration, while higher molarities produced greater instability in film formation upon drying. Table 1 presents a detailed description of the spectral peaks considered important to this analysis. A representative Raman spectrum for a desiccation solution having 1.2 M trehalose-water solution is shown in Fig 2A. Fig 2B defines the correlation curve used to relate the spectral ratios with the water content. The total average of the four drawing conditions are presented in Fig 2C. While there is no significant statistical difference in residual water content of the samples desiccated using the drawing rates of 50, 100, and 150 mm/min, the 200 mm/min drawing rate produced much higher average water content (average difference of 0.08 g_H20_/g_DW_). The 100 mm/min full film dataset (Fig 2D) shows very little variation of water content (0.2085 ± 0.01974 g_H20_/g_DW_) across the full film length of the coated substrate, indicative of high spatial uniformity. High uniformity was also observed for the 50 mm/min condition, while the 150 and 200 mm/min conditions displayed greater instability in film evolution.

**Table 1:** Significant Raman bands for water-trehalose solution.

Peak designation	Wavenumber (rel. 1/cm)	Bond assignment
a	852	δ(OCH)[20]
b	923	δ(COH)[20]
c	2884	ν(CH2)sy
d	2940	ν(CH2)as
e	3220	ν(OH)sy
f	3430	ν(OH)as

The peak designations are related to the representative Raman spectra of a water-trehalose solution, shown in Fig 2A. Key for bonding modes; δ: bending, ν: stretching, as: asymmetric, sy: symmetric.

When the desiccation processed samples were placed in a desiccation box, the loss of moisture after two hours was minimal indicating that the surface tension mediated desiccation technique developed here is highly efficient in desiccating the samples (S1B Fig). By desiccating overnight in an oven under vacuum, it was seen that samples for the 100 and 150 mm/min conditions had non-significant changes in water content. The 50 mm/min drawing rate had an initial water content to dried water content ratio of less than one, possibly indicating atmospheric inclusion. The 200 mm/min condition had a ratio of greater than one, likely indicating that residual moisture was evaporated out of the film.

### Membrane integrity and cellular viability

**Fig 3:**
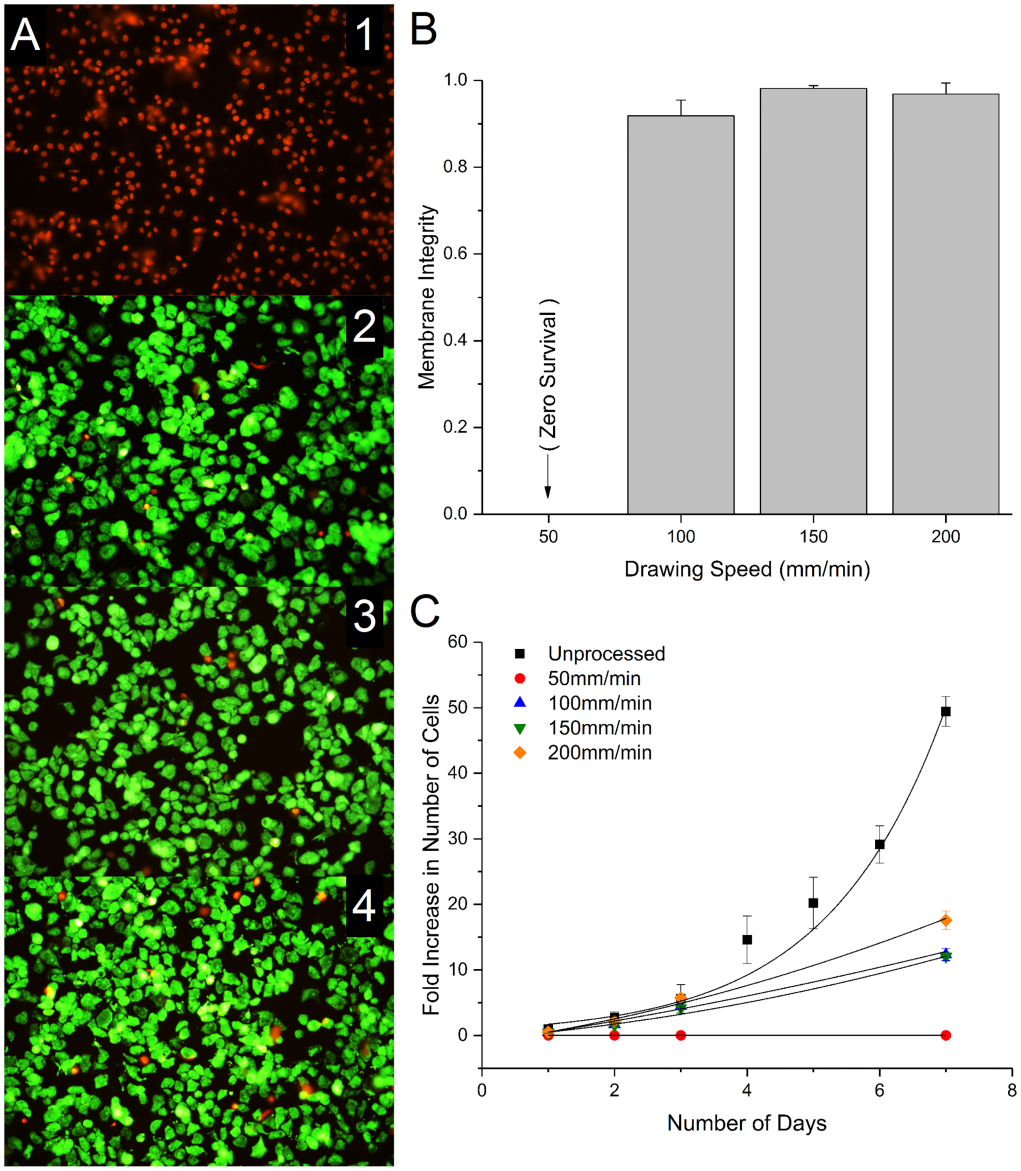
Cell viability. **A)** Optical images were acquired using Syto13/Ethidium Bromide fluorescent stains. 1–4 show in order; 50, 100, 150, 200 mm/min drawing rates, using a 1.2 M trehalose solution. The drawing rate dependence on cell survival is apparent and enumerated in **B**. **C)** Growth expressed as fold increase for 7 days after rehydration for the four processing conditions as well as a non-treated control group.

Post processing membrane integrity is quantified by fluorescent microscopy in Fig 3, panels A1-A4. Immediately after processing, cells were rehydrated with staining solution to physiological conditions as previously described. Fig 3B indicates there was no survival at the 50 mm/min drawing speed, but there was a marked improvement (>90%) in immediate viability of cells at drawing rates of 100 mm/min and greater. Cells were grown for seven days after processing and enumerated on days 1, 3, and 7 to quantify growth (Fig 3C). Steady growth (fold increase with respect to initial cell density) was observed for 100–200 mm/min conditions. The growth rates observed were less than an unprocessed control group, however.

### Metabolic profiles

**Fig 4:**
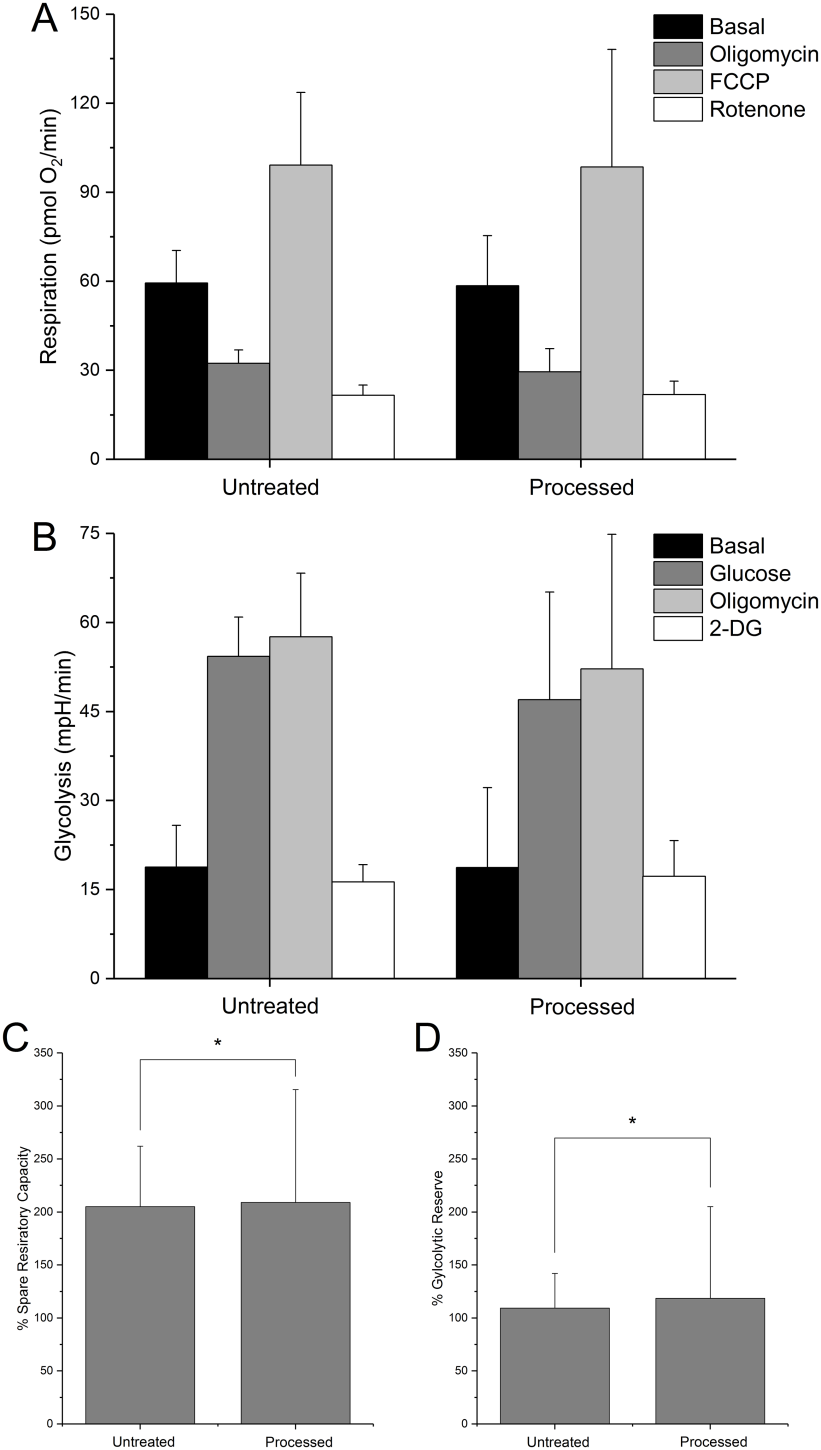
Metabolic characterization of dip coated cells. Bioenergetics were assessed with a Seahorse XFp for both untreated cells and cells which were processed via dip-coating technique (100 mm/min, 1.2 M trehalose solution), rehydrated immediately, and cultured for 14 days. **A)** Respiration (oxygen consumption rate) is shown, with legend titles referring to readings after specified reagent injections. **B)** Glycolysis (extracellular acidification rate) is shown similarly to A. **C)** Spare respiratory capacity is a (%) ratio between FCCP and Oligomycin groups in A. **D)** Glycolytic reserve is the (%) ratio between oligomycin and glucose groups in B. There was no statistical difference between treated and untreated groups. Error bars show +SD, n = 6.

Metabolic profiles of the dip-coated cells are presented in Fig 4. Processed cells used the 100 mm/min optimal condition and were immediately rehydrated and placed into aforementioned culture conditions. After 14 days, cells were collected and analyzed as described previously. Oxygen consumption rates provide for simple evaluations of respiration. Statistically equivalent basal OCRs were observed between untreated and processed cells (Fig 4A). Similar trends followed for the entire suite of metabolic conditions. Both groups showed equivalent responses to oligomycin suppression, FCCP uncoupled respiration, and rotenone-antimycin A inhibited respiration OCR measurements. Glycolysis, as measured by acidification rate, also showed negligible differences between processed and control groups (Fig 4B). Glycolysis stimulated by glucose saturation, followed by further stimulation by ATP inhibition by oligomycin injection, both had effects to increase ECAR, which was similar for both groups. Spare respiratory capacity is defined as $\frac{maximum\;respiration}{basal\;respiration}\times 100\%$ and is indicative of the ability of mitochondria to respond to an energy demand. Maximum respiration is taken as the highest OCR value after FCCP had been injected to the well. Basal respiration is the OCR value of non-induced cells. Similarly, glycolytic reserve is defined as $\frac{glycolytic\;capacity\;rate}{glycolysis}\times 100\%$ and describes the glycolytic response to cellular energy needs. Glycolytic capacity rate is the maximum ECAR value recorded after oligomycin injection, and glycolysis here means the highest ECAR value after being supplemented with glucose. There was no statistical difference between processed and live groups, considering both respiratory and glycolytic reserve, indicating that permanent intracellular damage is not incurred.

### Molecular analysis of changes in cell membrane composition

**Fig 5:**
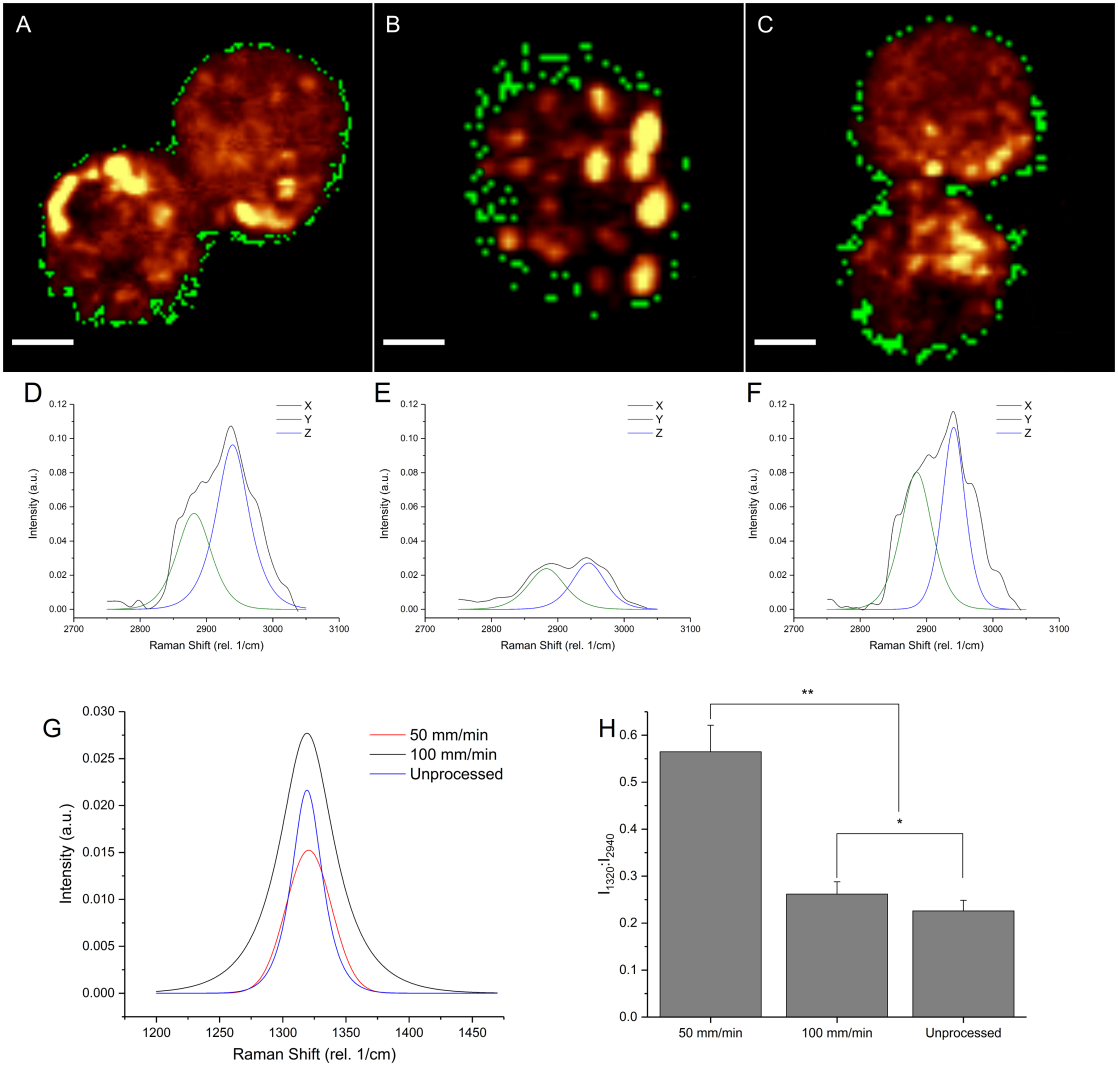
Raman hyperspectral analysis of membrane functionality. **A-C)** A mask (shown as green pixels) of cellular membrane spectral data was created to compare membrane functionality for dip coated cells. Scale bar = 5 μm. Cells are shown integrated on CH2 stretching band for spatial reference, with warmer colors indicating higher relative concentration. Cells were processed and then rehydrated immediately. Raman scanning was done on the third day after rehydration. The drawing rate of 100 mm/min **(C)** was compared to both non-treated cells **(A)** and cells processed at 50 mm/min **(B)**. Peak analysis consisted of deconvolution of CH2 stretching **(D-F)** [into Y: CH2sy and Z: CH2as peaks, shown with X: original smoothed spectra], and amide III **(G)** regions. **H)** The ratio of CH2 asymmetric (2940 rel. 1/cm) stretching peak intensity to amide III (1320 rel. 1/cm) peak intensity was calculated. Unprocessed cells and those at the optimal drawing rate had no statistical difference to each other (*), but both were significantly different to the 50 mm/min condition (**).

The effect of desiccation on the molecular components of the cell membrane was investigated using Raman microspectroscopy and hyperspectral imaging techniques. Spatially resolved spectral masks (shown as green pixels) representing signatures of the cell membranes obtained through hierarchal cluster analysis (HCA) were developed [21] for three different conditions as described above (unprocessed, Fig 5A; processed at 50 mm/min, Fig 5B; and processed at 100 mm/min, Fig 5C). Pseudo-colored hyperspectral maps integrated on CH2 stretching are shown in background in Fig 5A–5C to show cellular spatial definition. Fig 5D–5F show the deconvoluted CH2 symmetric and asymmetric stretching bands alongside the original smoothed spectra for unprocessed cells (5D), cells processed at 50 mm/min (5E), and those processed at 100 mm/min (5F). Fig 5G similarly shows deconvoluted spectra for the amide III peak for all three conditions.

Finally, taking the ratio of the CH2 asymmetric peak at 2940 rel. 1/cm and amide III at 1320 rel. 1/cm shows the strong correlation between unprocessed and 100 mm/min processed cell membrane characteristics, which are significantly different to the 50 mm/min processed cells.

## Discussion

Desiccated storage of biological samples is an alternative to low temperature preservation. Desiccated state storage is widely observed in nature among the cryptobiotic organisms and development of comparable biopreservation strategies can facilitate easy and economic transport of stored biologics including cells. In recent years, several lyoprocessing techniques have been developed for desiccated-state preservation of cells including freeze-drying [22], spray drying [23], and isothermal desiccation [24]. Freeze drying has been successfully implemented to stabilize germ cells [25] and platelets [22]. Isothermal drying techniques have been extensively studied due to their operational simplicity over techniques such as freeze drying and spray drying [13]. Fast lyoprocessing techniques have the added advantage of limiting the exposure time to non-physiological states during processing [11, 13]. The primary goal of this study was to develop a viable lyoprocessing technique that can be used to desiccate cells and cellular materials rapidly with high uniformity.

### Importance of fast processing in lyopreservation

For successful lyopreservation it is important for the cells to survive the process of desiccation with minimal injury. Fast lyoprocessing techniques that limit the duration of exposure to non-physiological hyperosmotic environments have been shown to improve survival in mammalian cells following desiccation processes when compared with passive slow drying techniques [11, 13, 26]. Reduction of cumulative osmotic stress in lyoprocessed cells has been suggested as one of the reasons for such improved outcomes and several fast drying techniques have been developed in recent years to lyoprocess mammalian cells including microwave drying [26], spray drying [23] and spin drying [13]. The surface tension based drying technique described here can produce a highly uniform vitrified matrix. In this study, we have demonstrated that cells attached to a suitable substrate can be encased in the vitrified matrix and the rate of drying can be modulated by varying the drawing rate of the substrate. The optimal dry processing rate for surface tension mediated drying process described here has a processing speed of 100 mm/min which takes 72 seconds to process one slide. When compared with the fast drying technique of spin-drying, both have similar processing time [13]. However, the technique described here is simpler from processing standpoint.

### Processing uniformity

While fast drying is an important factor in determining post-desiccation survival of cells in lyoprocessing, spatial heterogeneity of the desiccated matrix have also been hypothesized to play a crucial role in preventing loss of viability following desiccation [9]. Local non-uniformities in moisture content leads to differential molecular mobility in microenvironments which may degrade the entire sample while in storage [27]. Desiccation processing cells using passive diffusion techniques [1, 11] create local non-uniformities in residual moisture contents in the dried product due to several factors including non-uniform surface evaporation [28], Marangoni flow instabilities [29], and interface phase separation of the solute [30, 31]. The surface tension based desiccation technique described here can be used to create highly desiccated films with very uniform moisture contents (Fig 2C). The uniformity of residual moisture content in the dried samples were comparable to processing uniformity obtained using the spin-drying technique.

It is important to mention that gravimetric analysis using micro-precision balances could not be reliably used to quantify the residual water contents in the samples dried using the surface tension mediated drying technique. Therefore, a high-resolution Raman spectroscopic technique was used to quantify localized water contents. The correlation shown in Fig 2B gives an excellent approximation (R^2^>0.99) of residual moisture content from Raman spectral intensity ratio. This allows for easy conversion of sensitive Raman spectral detection into otherwise indeterminate moisture change. Spatially resolved Raman microspectroscopic characterization of the residual moisture content in the desiccated samples here demonstrate high processing uniformity (Fig 2D).

HCA analysis of the hyperspectral imaging of the Raman signals were also used to investigate the relative positioning of the desiccated trehalose film on the dry processed cellular samples. Depth scanning yields an area scan in a vertical (XZ) plane. Such depth scans show that the trehalose films had consistent depth across the stable processed areas, including cellular regions. It follows that cells were fully enveloped in the trehalose film. A representative hyperspectral image (S2A Fig) shows the relative position of the cell (pseudo-colored blue) and trehalose film (pseudo-colored magenta) to demonstrate this. This indicates that the process results in encasing the cells in a trehalose matrix. Trehalose is known to form glassy skins when exposed to low moisture environment and this can entrap water under the glassy skin [32]. However, the intensity of the average spectral signature of the OH stretching band of water molecules in the trehalose film above the cellular material (S2A Fig) is almost identical to the OH stretching band from the cellular region underneath it (S2C Fig). This strongly suggests the uniformity in residual water content in the cell samples processed using the technique described here. Furthermore, placing the desiccated samples in a desiccation box upon processing for extended duration of time did not result in any appreciable change in average residual moisture content (data not shown).

### Cellular viability and metabolic output

Immediate membrane integrity following cellular processing can indicate the extent of cellular injury caused by this technique. Both the membrane integrity of the cells immediately following desiccation processing (Fig 4) and metabolic health (Fig 5) demonstrate the feasibility of this technique to lyoprocess mammalian cells. In this study, for lyoprocessing parameter optimization of drawing rate, it was found that processing at a moderate drawing rate (100 mm/min) produce exceptional outcomes in terms of membrane integrity and survival. Quantification of membrane integrity using standard fluorescence microscopy techniques following desiccation processing the cells using the drawing rate of 100mm/min indicate more than 90% membrane integrity in the cell samples. This is a marked improvement over previous desiccation studies that uses passive slow drying technique where only 70% of membrane integrity has been reported [1]. However, the results are comparable to the fast-drying techniques such as spin drying. The drawing rate of 100 mm/min was considered as an optimum processing parameter for the current study because of low average residual moisture content while the cell samples maintained high immediate membrane integrity following rehydration.

A detailed investigation of cellular energetic characteristics of the cells processed using 100 mm/min indicated no appreciable difference in metabolic properties (Fig 4) when compared with the control cells that were not desiccated. Respiration and glycolysis showed no significant decline after processing and rehydration. These results respectively indicate that the mitochondria and overall cell energy producing mechanisms are left unadulterated following desiccation processing. The energy potentials shown are encouraging for the lyoprocessing technique described before.

Long term growth following lyoprocessing indicates the propensity for cellular proliferation. Comparing with an unprocessed control group shows the growth rate to be about halved, but still growing steadily. While the bioenergetics of the cell were found to be unaffected, potential for growth was diminished. This reduced capacity may be due to residual chemical triggers left by the lyoprocessing impetus. This may be improved by intracellular stabilizers introduced into growth or desiccation medium, but is left for future studies.

Improvements in the dry processing outcomes may also depend on pre-processing strategies in addition to the cellular density parameters. There is also a significant scope of improvement considering the pre-processing strategies involved that can be used to ameliorate processing stresses in the cells. Here fluid phase endocytosis was used to introduce trehalose in the intracellular space. While it has been demonstrated that trehalose plays an important role in protecting the cells from desiccation stresses when present on both sides of the cell membrane [33], in recent years several other protective molecules have been identified including late embryogenesis abundant (LEA) cells that can help improving the outcome of the dry processed cellular samples [17].

### Raman microspectroscopic estimation of membrane damage

Changes in membrane composition and related injury have a strong impact in survival of the desiccated mammalian cells [11, 33]. Desiccated membrane proteins are known to form aggregates resulting in significant changes in membrane geometry and constituent molecular structure, such as hexagonal holes which can impair the overall integrity of the cellular membrane [32, 33]. Combined HCA analysis along with hyperspectral imaging techniques using spatially resolved Raman microspectroscopic signal was used to characterize and detect changes in membrane structure upon desiccation (Fig 5). The analysis supports the conclusion that cells processed at 100 mm/min and unprocessed groups were functionally similar, while both were significantly different from the cells at 50 mm/min drawing rate which demonstrated poor membrane integrity upon rehydration (Fig 3A). This analysis provides a mechanistic outlook to the membrane injury which can be used to evaluate and predict the outcome of a desiccation technique. The ratio seen in Fig 5H indicates the ratio of amide III functional groups present per spectral pixel concentration of the organic molecules. Since only the positional area corresponding to the cell membrane is considered, it indicates the drying rate-effect induced collapse of amide bonds in the cell membrane, which is indicative of injury.

Continuing development and optimization of the surface tension based drying technique described is a viable technique to lyoprocess mammalian cells. The technique is capable of desiccating cell samples attached to a substrate rapidly and uniformly. The cellular injury during processing can be minimized by changing the processing parameters that are easy to modulate and automate. While the current study focuses on the drying aspect only, significant work needs to be done to store the desiccated sample at non-cryogenic or ambient temperatures. Based on the stability requirement during storage, the processing parameters developed in the technique developed here can be modulated to suit a wide range of biologics for lyoprocessing.

## Supporting information

S1 Fig Relative water retention of processed films. Quartz plates were processed and immediately scanned by Raman. They were dried by drierite (2 hr) and vacuum oven (18 hr) and scanned after both drying steps. Taking a ratio of the OH stretching (~3300 rel. 1/cm) band to the CH2 stretching (~2940 rel. 1/cm) band gives an estimate of water to dry weight. Comparing spectral ratios of initial and dried conditions shows that dry box had marginal effects on water content and vacuum drying for the 100 and 150 mm/min conditions. The 50 mm/min condition had an apparent increase in moisture content after baking, while the 200 mm/min condition lost residual moisture. A ratio of 1 indicates no moisture change upon drying.
(TIF)

S2 Fig Depth scan of HepG2 cell processed in trehalose film. A) Depth scan separated by HCA analysis, directionally, orange being air and brown being glass plate. B) Polarized light micrograph of cells in coating. Line 1–2 shows scanning position of A. C) Raman spectra color matched to A. The cell is predominantly in blue region and the trehalose coating is magenta.
(TIF)
