# Supplementary figures and images for: Development of a surface tension mediated technique for dry stabilization of mammalian cells
Source: PLoS One. 2018 Mar 5;13(3):e0193160. doi: 10.1371/journal.pone.0193160 (PMC5837090; doi:10.1371/journal.pone.0193160)

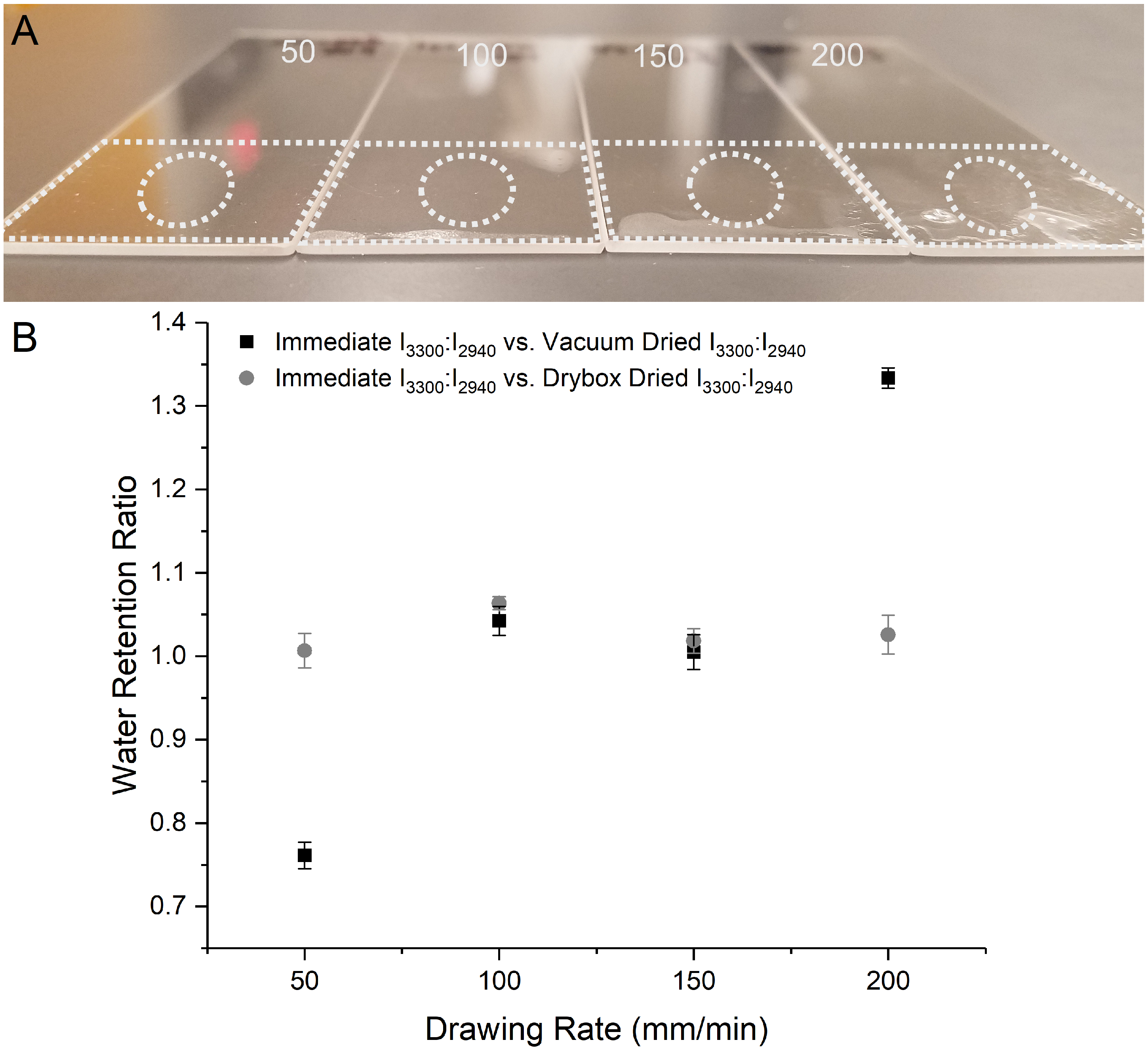

Supplement: S1 Fig — Quartz plates were processed and immediately scanned by Raman. They were dried by drierite (2 hr) and vacuum oven (18 hr) and scanned after both drying steps. Taking a ratio of the OH stretching (~3300 rel. 1/cm) band to the CH2 stretching (~2940 rel. 1/cm) band gives an estimate of water to dry weight. Comparing spectral ratios of initial and dried conditions shows that dry box had marginal effects on water content and vacuum drying for the 100 and 150 mm/min conditions. The 50 mm/min condition had an apparent increase in moisture content after baking, while the 200 mm/min condition lost residual moisture. A ratio of 1 indicates no moisture change upon drying. (TIF) [file pone.0193160.s001.tif]

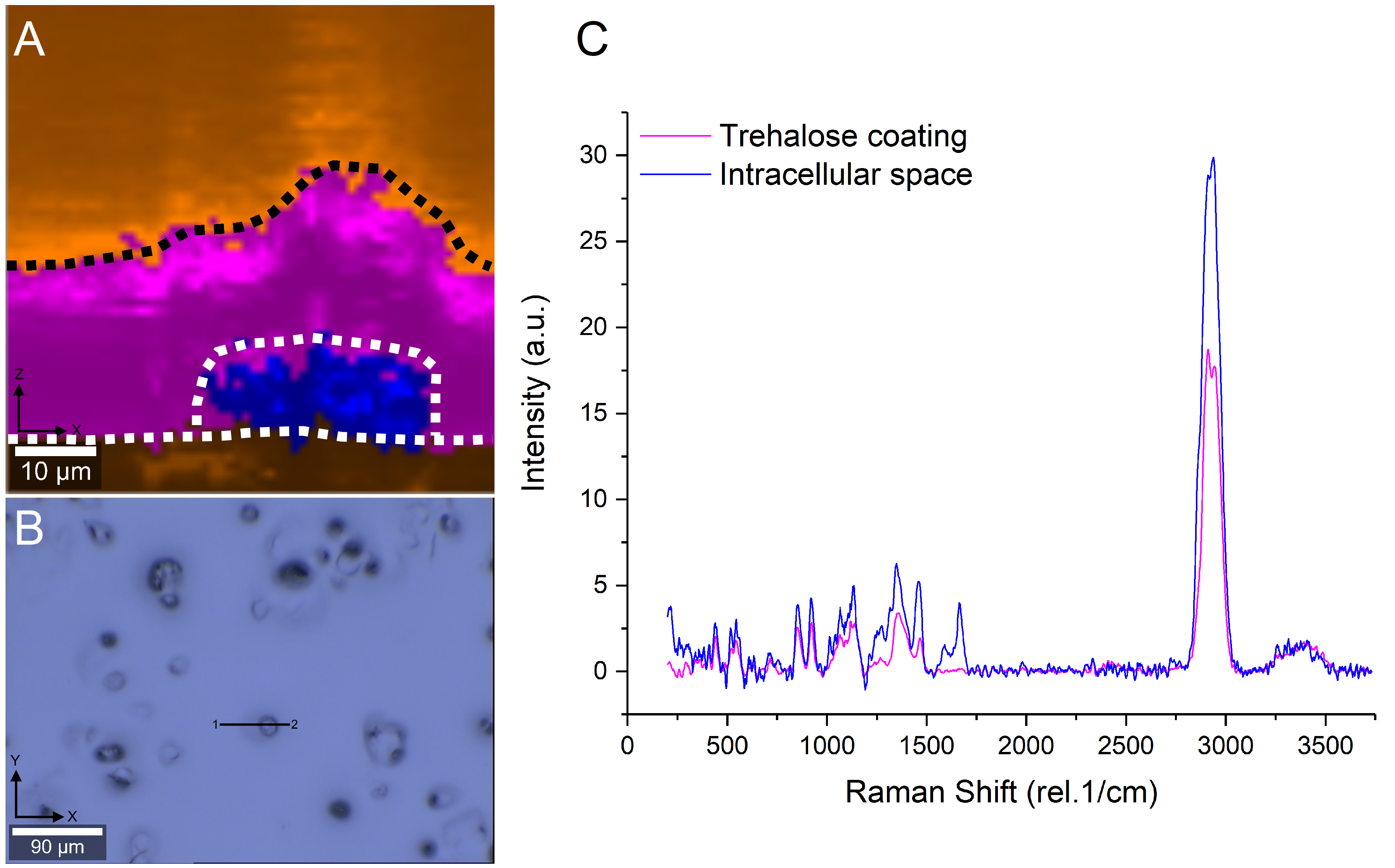

Supplement: S2 Fig — A) Depth scan separated by HCA analysis, directionally, orange being air and brown being glass plate. B) Polarized light micrograph of cells in coating. Line 1–2 shows scanning position of A. C) Raman spectra color matched to A. The cell is predominantly in blue region and the trehalose coating is magenta. (TIF) [file pone.0193160.s002.tif]
